# TACC3 transcriptionally upregulates *E2F1* to promote cell growth and confer sensitivity to cisplatin in bladder cancer

**DOI:** 10.1038/s41419-017-0112-6

**Published:** 2018-01-22

**Authors:** Zhi-Rui Lin, Meng-Yao Wang, Shi-Yang He, Zhi-Ming Cai, Wei-Ren Huang

**Affiliations:** 1 State Engineering Laboratory of Medical Key Technologies Application of Synthetic Biology, Shenzhen Second People’s Hospital, The First Affiliated Hospital of Shenzhen University, Shenzhen, 518039 China; 2 State Key Laboratory of Oncology in South China, Collaborative Innovation Center for Cancer Medicine, Sun Yat-sen University Cancer Center, Guangzhou, 510060 China; 3 Radiation Oncology Department, Affiliated Cancer Hospital & Institute of Guangzhou Medical University, Guangzhou, 510060 China

## Abstract

Accumulating evidence has shown that transforming acidic coiled-coil 3 (TACC3) is deregulated in a broad spectrum of cancers. In the present study, we reported that TACC3 was markedly elevated in bladder cancer, especially in muscle-invasive bladder cancers (MIBCs). The upregulation of TACC3 was positively associated with tumor invasiveness, grade, T stage, and progression in patients with bladder cancer. Furthermore, a Kaplan–Meier survival analysis showed that patients with bladder cancer whose tumors had high *TACC3* expression experienced a dismal prognosis compared with patients whose tumors had low *TACC3* expression. Functional studies have found that TACC3 is a prerequisite for the development of malignant characteristics of bladder cancer cells, including cell proliferation and invasion. Moreover, TACC3 promoted G1/S transition, which was mediated via activation of the transcription of *E2F1*, eventually enhancing cell proliferation. Notably, the overexpression of TACC3 or E2F1 indicates a high sensitivity to cisplatin. Taken together, these findings define a tumor-supportive role for TACC3, which may also serve as a prognostic and therapeutic indicator in bladder cancers

## Introduction

According to the latest cancer statistics, bladder cancer is the ninth most-common cancer worldwide and the fourth major cause of morbidity and mortality in men^1^. In China, the incidence rate of bladder cancer has continued to rise. Bladder cancers are considered either low-grade non-muscle-invasive bladder cancers (superficial or NMIBCs), which account for 70% of tumor occurrence, or high-grade muscle-invasive bladder cancers (invasive or MIBCs), which are responsible for the majority of bladder cancer-specific deaths. Although its 5-year survival rate is around 90%, NMIBCs are associated with a high rate of recurrence, and 5–30% of these cases will progress to MIBCs, which have a relatively poor 5-year overall survival (68% for T2, which decreases to 15% for pT3 and pT4)^2^. Radical cystectomy with perioperative cisplatin-based combination chemotherapy is the predominant therapeutic regimen for high-risk MIBCs. However, cisplatin-based chemotherapy is only effective in 30–40% of cases, and it is not yet possible to prospectively identify the patients who are likely to experience a benefit^3^. Therefore, there is an urgent need to identify promising biomarkers that predict a higher risk for disease progression and that inform clinical intervention more precisely.

The transforming acidic coiled-coil protein 3 (TACC3) protein belongs to the TACC family of centrosomal proteins with a highly conserved C-terminal coiled-coil domain^4^. TACC3 is one of the essential regulators of mitotic progression and functions via the promotion of microtubule (MT) stability during the assembly of mitotic spindle in mammals^5–8^. Moreover, the aberrant overexpression of TACC3 has been found in many different types of tumors^9–11^. Remarkably, in bladder^12–14^ and other solid cancers^15–19^, *TACC3* may fuse to *FGFR3* by chromosomal arrangement, which results in kinase continuous activation, such that it is sufficient for transformation and associated with progression. In addition, several Genome-wide association study (GWAS) have identified *TACC3* as a candidate bladder cancer susceptibility gene^20–22^. However, the potential role of TACC3 and its molecular mechanisms in bladder cancer remain to be addressed.

In the current study, we found that TACC3 expression levels are significantly elevated in bladder cancer, and more importantly that they are positively correlated with tumor progression and poor prognosis. In addition, we confirmed the oncogenic role of TACC3 in bladder cancer through a series of functional tests. Furthermore, we determined that overexpression of TACC3 promotes G1/S transition via the transcriptional activation of *E2F1*. Importantly, cancer cells that express high levels of TACC3 or E2F1 display increased sensitivity to cisplatin. Thus, our results demonstrate a novel function of TACC3 in the regulation of the cell cycle, which contributes to tumorigenesis. TACC3 may therefore serve as a potential prognostic and diagnostic indicator and as a therapeutic target in bladder cancer.

## Results

### TACC3 is markedly upregulated in bladder cancer and is strongly associated with invasive cancer and a dismal prognosis

**Fig. 1:**
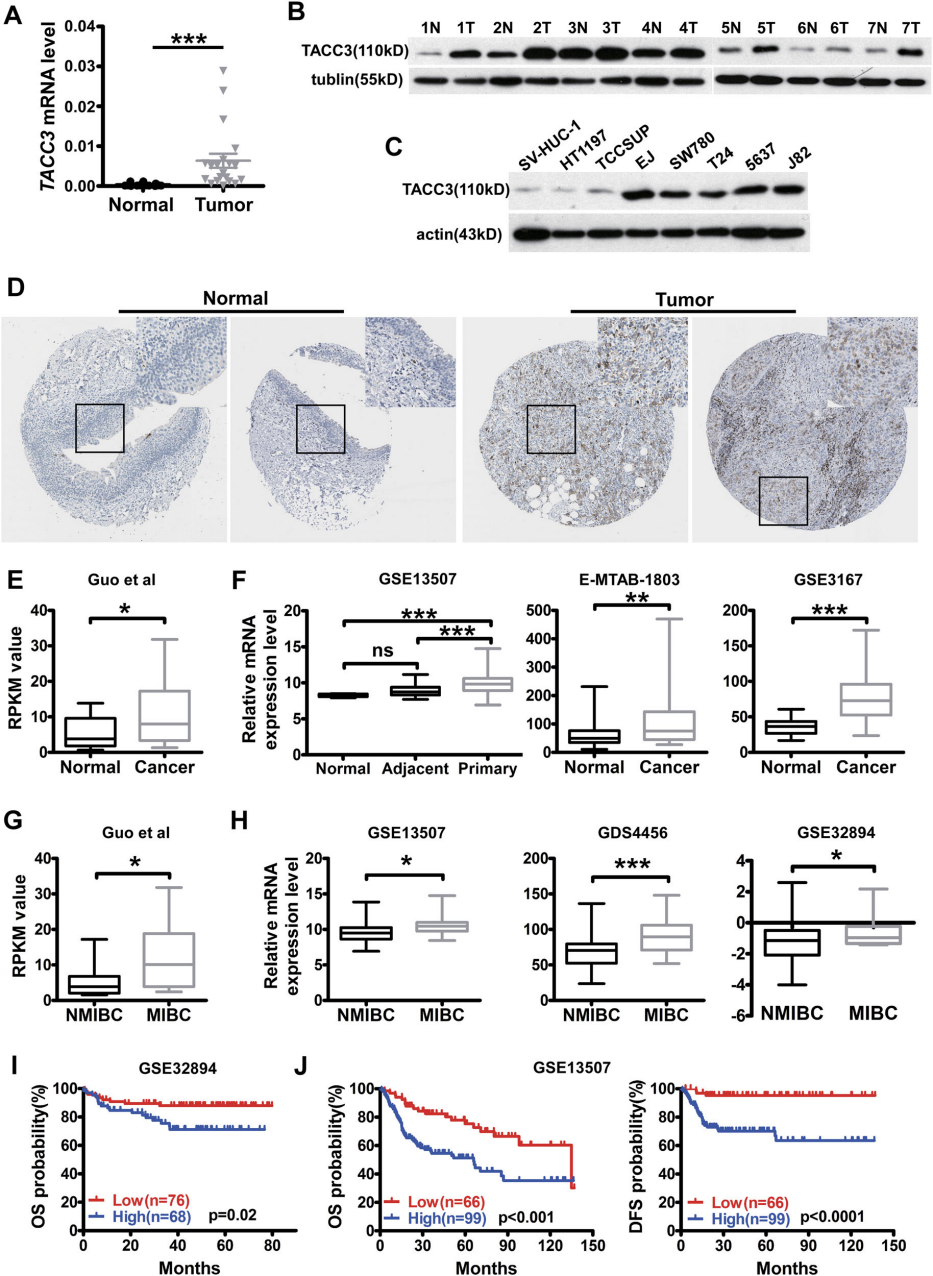
TACC3 is markedly upregulated in bladder cancer **a** QPCR analysis of TACC3 mRNA expression in clinical specimens from bladder cancer patients (normal = 12, tumor = 20). **b** Western blotting analysis of TACC3 protein expression in seven pairs of matched bladder cancer tissues (T) and adjacent normal tissues (N). **c** Western blotting analysis of TACC3 expression in seven human bladder cancer cell lines including HT1197, TCCSUP, EJ, SW780, T24, 5637, and J82 and one immortalized bladder epithelial cells SV-HUC-1. **d** Representative images of negative TACC3 IHC staining in normal bladder tissues and representative images of strong TACC3 IHC staining in bladder cancer tissues. All images were downloaded from the Human Protein Atlas (www.proteinatlas.org). **e**, **f** Box plots of TACC3 expression levels in bladder cancer patients. TACC3 mRNA expression levels were downloaded from public data sets (Guo et al., GSE13507, GSE3167 from NCBI GEO; E-MTAB-1803 from EMBL-EBI). **g**, **h** The TACC3 mRNA expression levels were significantly upregulated in the MIBC subtype compared with the NMIBC subtype according to other GEO data (Guo et al., GSE13507, GDS4456, GSE32894)^14^. **i**, **j** Kaplan–Meier plots show the inverse association of the TACC3 mRNA level and the survival of patients with bladder cancer according to the GSE32894 and GSE13507 data sets (*p*-values were calculated by log-rank test). The expression difference between two groups was determined by the Student’s *t*-test. *p < 0.05, **p < 0.01, ***p < 0.001

We initially examined *TACC3* expression in bladder cancer tissues and in normal bladder epithelium by Quantitative Polymerase Chain Reaction (QPCR). As shown in Fig. 1a, tumor tissues had a more abundant level of *TACC3* mRNA than the normal epithelium. In addition, western blotting results showed that the level of TACC3 protein was robustly upregulated in urinary bladder cancer compared with paired adjacent non-tumor tissues and in most of the bladder cancer cell lines tested compared with the immortalized uroepithelial cells SV-HUC-1 (except HT1197 and TCCSUP cells) (Fig. 1b, c). Likewise, we analyzed the expression of TACC3 protein in clinical specimens from the Human Protein Atlas (www.proteinatlas.org) and found that TACC3 was strongly expressed in bladder cancers but weakly expressed in normal samples (Fig. 1d). To further investigate the upregulation of TACC3, we performed a data analysis of public data sets^14,23–25^, which indicated that *TACC3* expression was significantly elevated in tumor samples compared with their normal counterparts (Fig. 1e, f). Surprisingly, we noticed that the MIBC subtype exhibited a more profound increase in *TACC3* expression among the four different patient cohorts examined^14,23,26–28^ (Fig. 1g, h), which clearly indicated that the activation of *TACC3* might be a crucial genetic event in the development or progression to MIBC. In addition, high *TACC3* expression was significantly associated with gender (*p* = 0.013), invasiveness (*p* < 0.001), tumor grade (*p* < 0.001), tumor progression (*p* = 0.001), and T status (*p* < 0.001) in bladder cancer patients (Table 1). Moreover, the clinical significance of high *TACC3* expression was further assessed by a Kaplan–Meier survival analysis, which indicated the significant association between higher *TACC3* expression and poor outcome in two separate cohorts^23,24,28^. Taken together, these observations suggested that altered TACC3 expression might be instrumental in the initiation or progression of bladder cancers.

**Table 1:** Association of TACC3 expression with clinicopathological characteristics of bladder cancer patients in the Lee et al. cohorts

Characteristics	No. of patients	TACC3 expression		*p*-value
		Low	High	
All patients	165	66	99	
Age				
Median	66			
Range	24–88			
⩽66	84	39	45	
>66	81	27	54	0.086
Gender				
Female	30	6	24	
Male	135	60	75	0.013
Invasiveness				
NMIBC	103	55	48	
MIBC	62	11	51	<0.001
Grade				
Low	105	61	44	
High	60	5	55	<0.001
Progression				
No	134	62	72	
Yes	31	4	27	0.001
pT stage				
Ta	23	16	7	
T1	80	39	41	
T2–T4	62	11	51	<0.001

*p*-Value was calculated by Pearson's *χ*^2^ test

### TACC3 is essential for the malignant phenotype of bladder cancer

**Fig. 2:**
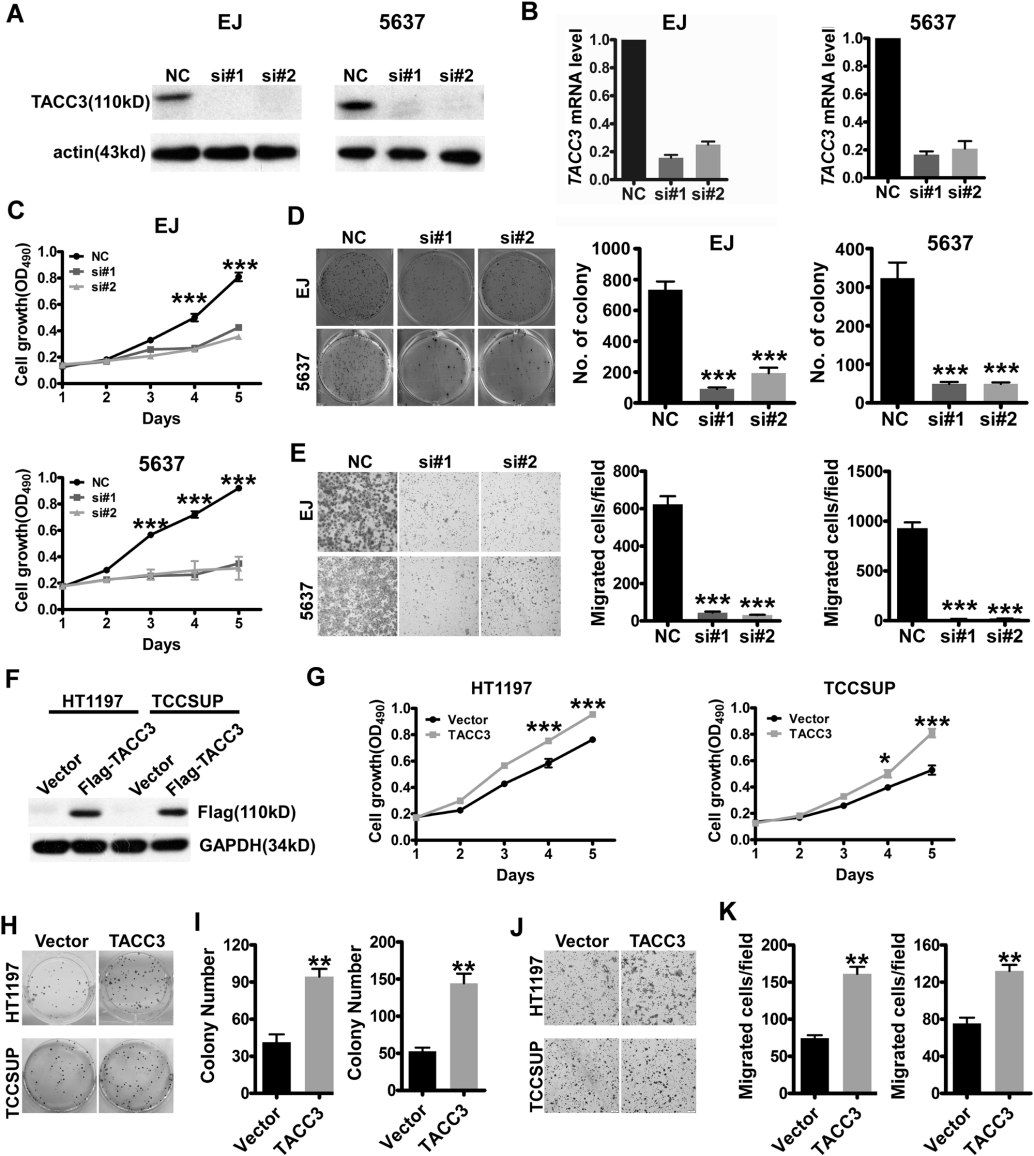
Alternative expression of TACC3 regulates cell growth and invasion in bladder cancer **a**, **b** Western blotting (**a**) and QPCR (**b**) analyses show almost complete elimination of TACC3 expression after TACC3 siRNA knockdown in EJ and 5637 cells. **c** Suppression of TACC3 in EJ and 5637 cells dramatically reduced their proliferative ability, as determined by MTT assay. ***p < 0.001 **d** TACC3 knockdown in EJ and 5637 cells markedly reduced their ability to form foci, as determined by the foci-formation assay. Left: representative images of colony formation. Right: error bars present means ± SEM (*n* = 3). *p*-Values were calculated by one-way ANOVA. **p < 0.01. **e** Transwell assay showed downregulated TACC3 reduced cell invasiveness. Left: representative images. Right: error bars present means ± SEM (*n* = 3). *p*-Values were calculated by one-way ANOVA. ***p < 0.001. **f** Levels of ectopically expressed TACC3 in HT1197 and TCCSUP cells were examined by western blotting. **g** Overexpression of TACC3 promoted cell growth, as shown by MTT assay. ***p < 0.001. **h**, **i** Overexpression of TACC3 increased the formation of foci, as determined by the foci-formation assay. **j**, **k** Overexpression of TACC3 enhanced cell invasiveness. The statistical results in **i** and **k** are reported as mean ± SEM. *p-V*alues were calculated by the Student’s *t*-test. **p < 0.01

To investigate the biologic activities of TACC3 in bladder cancer, we first examined the effect of siRNA-induced reduction of TACC3 by western blot (Fig. 2a) and Real-time Quantitative Polymerase Chain Reaction qRT-PCR (Fig. 2b). MTT assays showed that the knockdown of TACC3 dramatically inhibited the growth of both EJ and 5637 cells in vitro (Fig. 2c) and the growth of EJ cells in vivo (Supplementary Fig. 1). In agreement with this, the efficiency of foci formation was significantly reduced (Fig. 2d). Moreover, both EJ and 5637 cells displayed a large decrease in invasion after TACC3 was silenced (Fig. 2e). Conversely, ectopic expression of TACC3 (Fig. 2f) enhanced proliferation and growth in cancer cells compared with control cells according to MTT assays (Fig. 2g) and foci-formation assay (Fig. 2h, i). In addition, TACC3 overexpression increased the invasive properties of cancer cells (Fig. 2j, k). In general, these data indicated that TACC3 is strongly related to tumor progression.

### Regulation of the G1/S transition by TACC3

**Fig. 3:**
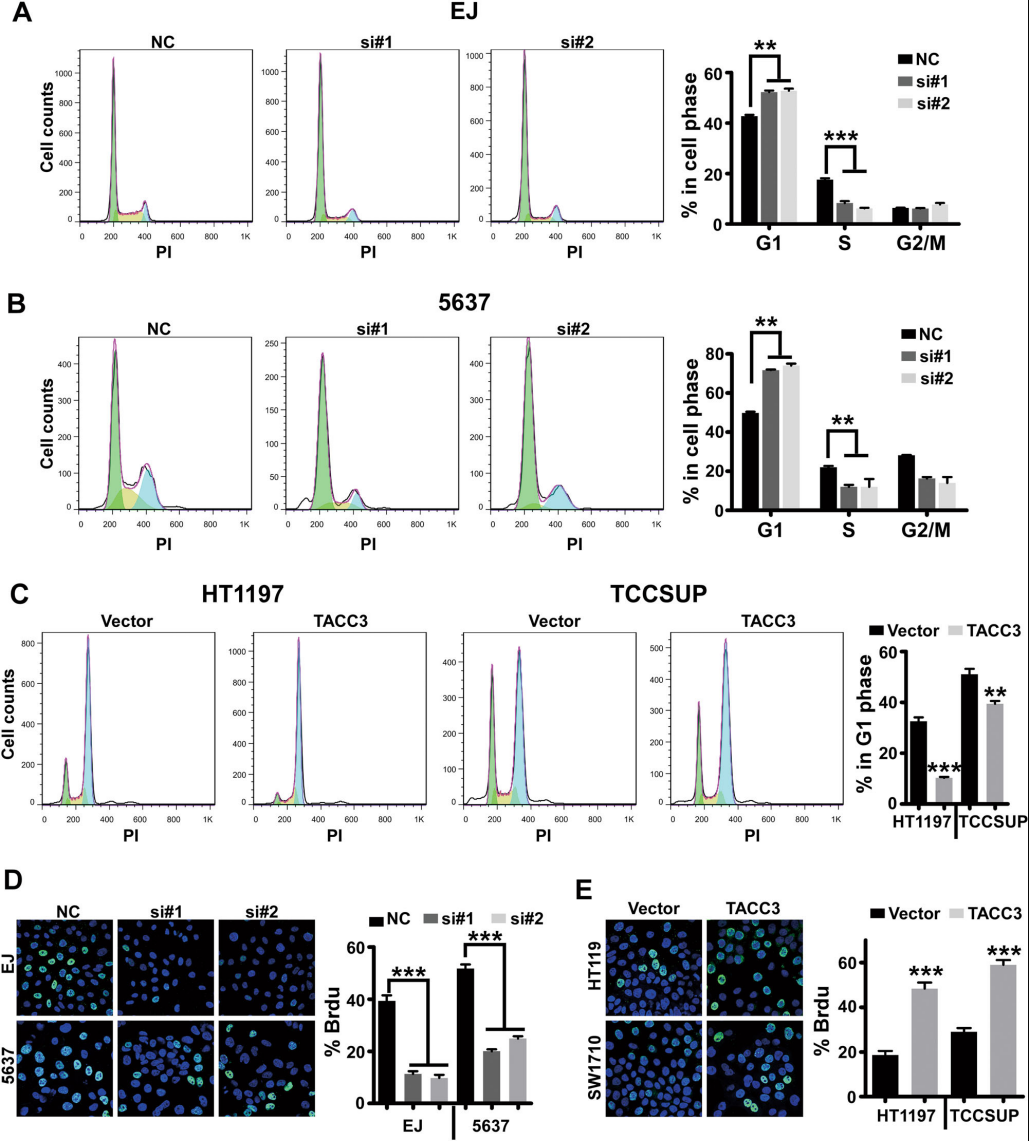
Regulation of the G1/S transition by TACC3 **a**, **b** EJ or 5637 cells were transfected with NC or TACC3 siRNA for 48 h and were then stained with propidium iodide and analyzed by flow cytometry (*n* = 3). **c** Ectopic expression of TACC3 in both HT1197 and TCCSUP cells decreased the percentage of cells in G1 phase in the presence of nocodazole (100 ng/ml) for 16 h (*n* = 3). **d** Repression of TACC3 inhibited DNA synthesis, which led to a decrease in the number of cells in S phase as shown by BrdU incorporation assay. **e** Overexpression of TACC3 promoted DNA synthesis, which led to an increase in the number of cells in S phase as shown by BrdU incorporation assay. Error bars represent mean ± SEM. **p < 0.01, ***p < 0.001

It is well known that abnormal alterations in the cell-cycle profile fuel the unlimited growth of cancer cells^29^. To determine whether TACC3 is involved in cell-cycle progression, we analyzed cellular DNA content by flow cytometry upon TACC3 depletion. As shown in Fig. 3a, b, TACC3 knockdown in EJ and 5637 cells induced obvious G1 arrest, which was concurrent with a decreased percentage of cells in S phase. To further investigate whether TACC3 is a key component in G1/S transition, we measured the percentage of cells in G1 phase in cell lines with ectopic expression of TACC3 upon treatment with nocodazole, which arrests cells in M phase. As shown in Fig. 3c, the percentage of cells in G1 phase was markedly decreased by TACC3 overexpression, which clearly indicated that the upregulation of TACC3 significantly promoted G1/S transition. In addition, a bromodeoxyuridine (BrdU) incorporation assay showed that TACC3 repression inhibited DNA synthesis in cells in S phase (Fig. 3d), and conversely, TACC3 upregulation greatly facilitated DNA synthesis (Fig. 3e). Collectively, these results provided evidence that TACC3 contributes to G1/S phase progression in the cell cycle in bladder cancer cells.

### TACC3 functions as a positive regulator of E2F1 in bladder cancer

**Fig. 4:**
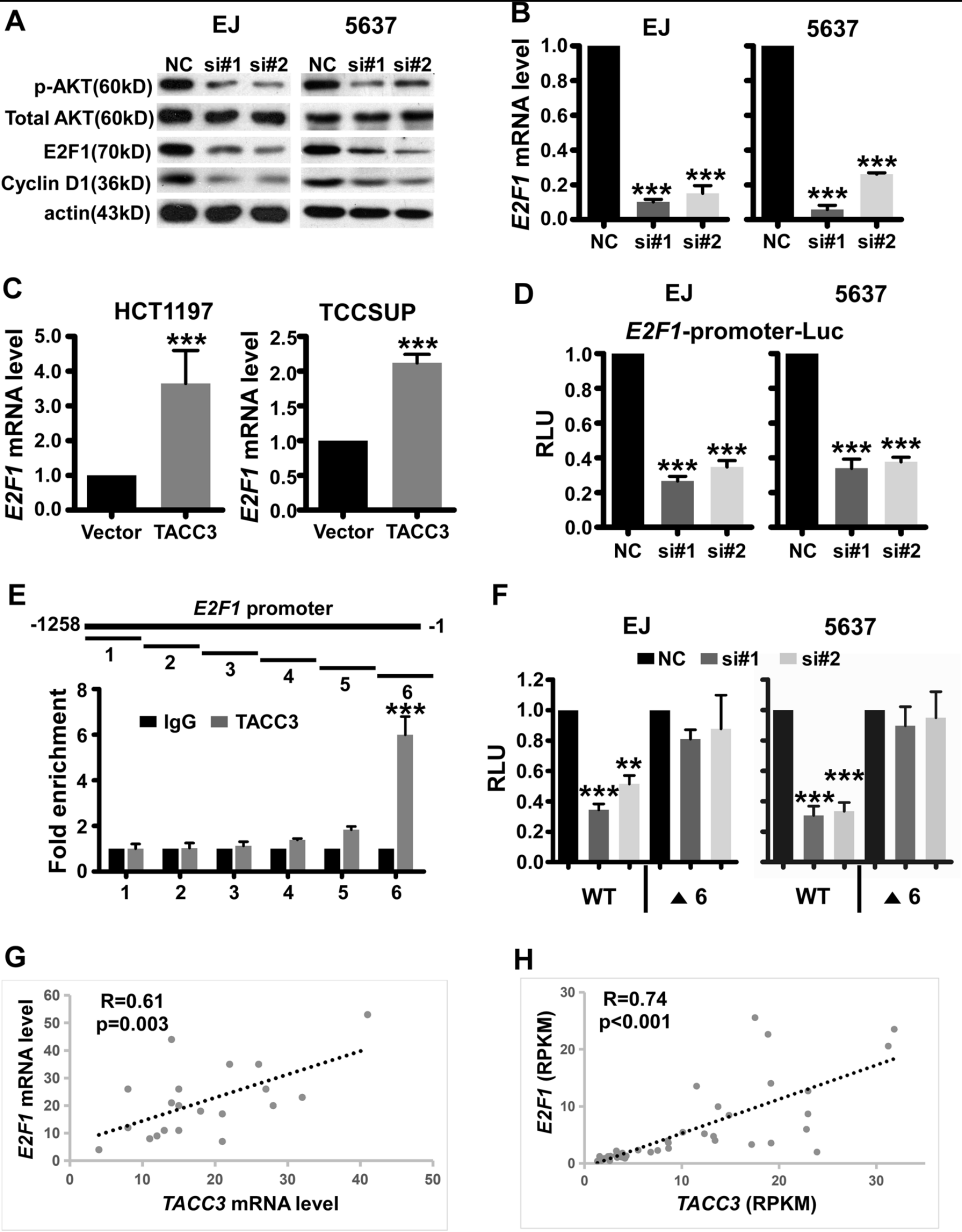
TACC3 positively regulates the expression of E2F1 **a** Western blotting revealed that TACC3 knockdown in EJ and 5637 cells caused a decrease in *P*-AKT, E2F1, and cyclin D1 protein expression. **b** QPCR revealed that TACC3 knockdown in EJ and 5637 cells repressed *E2F1* mRNA expression. Data are presented as mean ± SEM (*n* = 3). ***p < 0.001 in one-way ANOVA. **c** QPCR demonstrated that TACC3 overexpression upregulated the level of *E2F1* mRNA. Data are presented as mean ± SEM (*n* = 3). ***p < 0.001 in the Student’s *t*-test. **d** EJ or 5637 cells were first transfected with siTACC3 or control siRNA. After 36 h, the cells were co-transfected with the pGL3-E2F1 gene promoter luciferase or the pRL-TK Renilla luciferase construct, and 36 h later, the luciferase activity of the *E2F1* promoter was measured and the fold enrichment relative to the control siRNA was determined. Data are presented as mean ± SEM (*n* = 3). ***p < 0.001 calculated by one-way ANOVA. **e** Schematic representation of the *E2F1* promoter. The region of primers (Supplementary Table 2) used for the ChIP-qPCR assays is indicated. Bar graphs show the fold enrichment relative to IgG. Data are presented as mean ± SEM (*n* = 3). ***p < 0.001 calculated by the Student’s *t*-test. Fragment 1(-1258--1077), Fragment 2(-1076--809), Fragment 3(-808--551), Fragment 4(-550--404), Fragment 5(-403--221), and Fragment 6(-220--1). **f** Cells were first transfected with NC or TACC3 siRNA for 36 h. Then, cells were transfected with the luciferase reporter vector containing the E2F1 promoter or the Δ6 mutant. After 24 h of transfection, the cells were lysed, and the luciferase activity was measured. Data are presented as mean ± SEM (*n* = 3). *p*-Values were calculated by one-way ANOVA. **p < 0.01, ***p < 0.001. **g**, **h**, Graphs show a positive correlation between *TACC3* and *E2F1* mRNA levels in bladder cancer cell lines (Pearson’s *r* = 0.61, *p* = 0.003) and in bladder cancer clinical tissues (*n* = 40, Pearson’s *r* = 0.74, *p* < 0.001) based on RNA-seq data from a public database

Since our results suggested that TACC3 has a role in the transition from G1 to S phase in bladder cancer cells, we performed a western blot to analyze the expression of key cell-cycle regulatory proteins after modulation of TACC3 expression. As shown in Fig. 4a, TACC3 knockdown diminished the expression of p-AKT, E2F1, and cyclin D1.

It has been established that E2F1 is a master regulator of the cell cycle that promotes G1/S transition via the transactivation of a variety of genes involved in DNA synthesis and G1/S-regulatory genes, for example, *MCM4*^30^, *CDC6*^31^, *CDC25A*^32^. More importantly, previous studies identified E2F1 as a key mediator in the progression of NMIBC to MIBC. Thus, we hypothesized that E2F1 may participate in the TACC3-mediated deregulation of the cell cycle and disease progression and may be responsible for the aggressive characteristics of bladder cancer. We found that a repressed level of TACC3 decreased the expression of *E2F1* mRNA and that an elevated level of TACC3 increased the expression of *E2F1* mRNA, which verified the molecular mechanisms by which TACC3 promotes E2F1 expression (Fig. 4b, c). In addition, as illustrated in Fig. 4d, luciferase reporter assays showed that TACC3 was required for the transcriptional activity of *E2F1* promoter via knockdown of TACC3. Next, we performed ChIP-qPCR experiments to determine whether TACC3 can bind to the *E2F1* promoter. As shown in Fig. 4e, TACC3 was associated with the *E2F1* promoter in a region between nucleotides −220 and −1 (Fragment 6) from the transcription start site. Moreover, deletion of this region in the *E2F1* promoter abolished the TACC3 depletion-mediated downregulation of *E2F1* promoter activity (Fig. 4f), which demonstrated that the region that spans −220 to −1 is necessary for the regulation of *E2F1* expression by TACC3. Furthermore, we examined the mRNA expression of *TACC3* and *E2F1* in public data sets including expression data of bladder cancer cell lines from Cancer Cell Line Encyclopedia (CCLE)^33^, RNA-seq data from the study by Guo et al. and found a positive correlation between *TACC3* and *E2F1* mRNA level (Fig. 4g, h). Importantly, the mRNA expression of *MCM4*, *CDC6*, and *CDC25A*, which are the direct target genes of E2F1, was significantly downregulated after TACC3 silencing in EJ cells as well as significantly upregulated after TACC3 overexpression in TCCSUP cells. In addition, *TACC3* is highly co-expressed with *MCM4*, *CDC6*, and *CDC25A*, indicated by correlation analysis using RNA-seq data of 42 bladder cancer clinical samples from our group published before (Supplementary Fig. 2). These data suggested that TACC3 functions as a transcriptional activator of *E2F1* in bladder cancer.

### Elevated levels of TACC3 or E2F1 render cells more sensitive to cisplatin

**Fig. 5:**
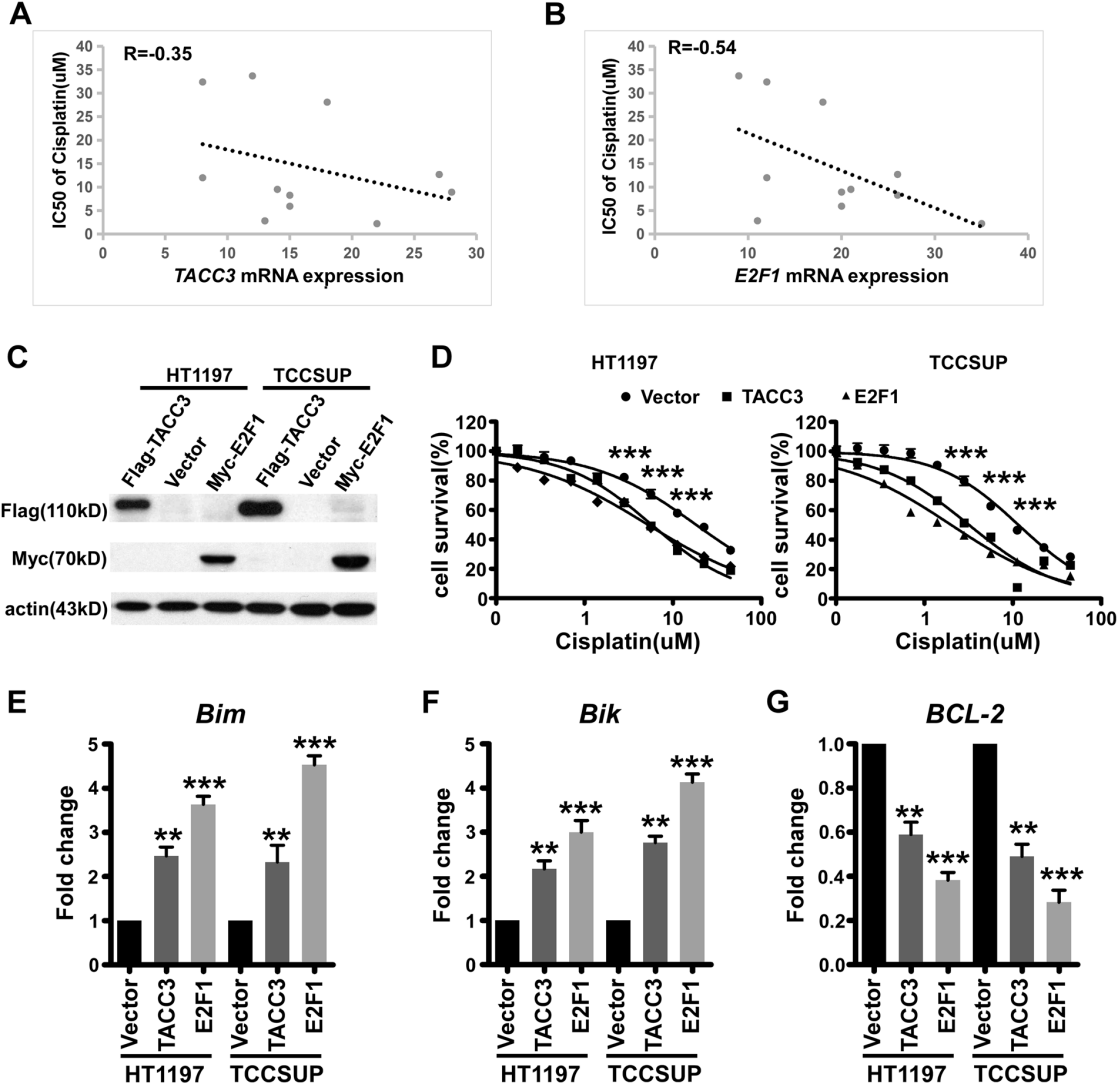
Elevated levels of TACC3 or E2F1 render cells more sensitive to cisplatin **a**, **b** Graphs show an inverse correlation between the IC50 of cisplatin and *TACC3* (*r* = −0.35) or *E2F1* (*r* = −0.54) mRNA levels in bladder cancer cell lines. **c** Western blot of transcriptionally overexpressed TACC3 or E2F1. **d** Cells that expressed high levels of TACC3, E2F1, or control vector were exposed to various concentrations of cisplatin for 96 h. Cell viability was measured by MTT assay. ***p < 0.001 determined by one-way ANOVA. **e**–**g** QPCR analysis of apoptosis-related genes including *Bik*, *Bim*, and *Bcl-2* in bladder cancer cell lines with exogenous expression of TACC3 or E2F1 after exposure to a high dose of cisplatin (10 µg/ml). Error bars represented mean ± SEM. **p < 0.01, ***p < 0.001 in one-way ANOVA

Since cisplatin-based chemotherapy is a classic treatment for bladder cancer, we wanted to elucidate the relationship of TACC3 expression and cisplatin sensitivity. Primarily, we conducted a correlation analysis between the *TACC3* mRNA level and the IC50 of cisplatin in bladder cancer cell lines using data downloaded from the CCLE. Unexpectedly, we found an inverse association between the *TACC3* mRNA level and the IC50 of cisplatin (Fig. 5a). In addition to the mRNA level of *TACC3*, the mRNA level of *E2F1* was also negatively correlated with cisplatin sensitivity (Fig. 5b). To validate this association, we established bladder cancer cell lines with exogenous expression of TACC3 or E2F1 (Fig. 5c), and as shown in Fig. 5d, the overexpression of TACC3 or E2F1 caused cellular hypersensitivity to cisplatin.

As is well known, E2F1 normally enhances cell-cycle progression, but it also induces apoptosis via the transcriptional upregulation of critical pro-apoptotic genes, such as *Bik* and *Bim*, and the transcriptional repression of distinct members of the anti-apoptotic protein family including *Bcl-2* under certain conditions, such as DNA damage and serum deprivation^34–41^. According to these findings, when exposed to a higher dose of cisplatin (10 µg/ml), bladder cancer cell lines with high TACC3 or E2F1 expression were both characterized by a strong transactivation response by *Bik* (Fig. 5e) and *Bim* (Fig. 5f), which paralleled a severe downregulation of the *Bcl-2* gene (Fig. 5g). These data suggested that elevated levels of TACC3 or E2F1 confer cells with sensitivity to cisplatin.

## Discussion

As a member of a family of three related proteins with highly conserved C-terminal coiled-coil domains, TACC3 is necessary for organization of mitotic spindle formation, which is required for proper chromosome segregation during cell division. In mitotic cells, phosphorylation by Aurora A drives the localization of TACC3 to centrosomes and the mitotic spindle^42–44^ where it can recruit ch-TOG to form a kinetochore fiber-stabilizing complex^45,46^. Knockdown of TACC3 leads to growth retardation and embryonic lethality. In addition, TACC3 is a prerequisite for progenitor cell expansion and hematopoiesis, which is not surprising given that its deletion triggers p53-mediated apoptosis^47^. Thus, the deregulation of TACC3 undoubtedly perturbs normal physiology and may even contribute to the development of human cancers. Indeed, TACC3 has been implicated in a wide range of human cancers including multiple myeloma, lung cancer, cervical cancer, ovarian cancer, and esophageal cancer^48–50^. Intriguingly, oncogenic fusion products between *FGFR3* and *TACC3* have been identified in glioblastoma, bladder, lung, and nasopharyngeal carcinomas. These oncogenic fusion products contain the C-terminal TACC domain of TACC3. To date, activating mutations^51,52^ and/or overexpression^53^ of FGFR3 are common in bladder cancers with low malignant potential and are associated with a lower risk of progression and better survival^54^. Nevertheless, the clinical significance and oncogenic potential of TACC3 in human urothelial carcinomas have not been elucidated. Herein, we demonstrate that TACC3 is upregulated gradually as bladder cancer progresses and that normal tissues exhibited the lowest expression of *TACC3*, followed by NMIBCs and MIBCs. Moreover, the overexpression of *TACC3* was significantly associated with invasiveness, grade, T stage, and progression, and higher *TACC3* expression predicted a worse overall survival and disease-free survival. Briefly, TACC3 is involved in the development of bladder cancer and may serve as a promising prognostic biomarker in this cancer type.

More recently, the oncogenic effects exerted by TACC3 during tumor progression have become clearer. Huang et al. confirmed the promoting role of TACC3 in the regulation of Epithelial-Mesenchymal Transition (EMT) and tumor growth. Zhou et al. reported that high expression of TACC3 enhances the stem cell-like phenotype of hepatocellular carcinoma (HCC) cells^55^. Li et al. showed that TACC3 could activate the Wnt/β-catenin signaling pathway to promote the metastasis of prostate cancer. In our present study, functional experiments illustrated that TACC3 depletion induces G1 arrest, decreases foci-formation ability, reduces cell growth in vitro and in vivo, and represses migration. Conversely, TACC3 overexpression elicits the opposite effect. These data support the emerging view that TACC3 is an important driving force in bladder cancer.

Increasing evidence suggests that TACC3 not only plays a critical role in cell division but it also functions as a transcriptional co-factor involving in gene transcription regulation. On the one hand, TACC3 can act as a co-repressor; for example, Garriga-Canut et al. revealed that TACC3 blocks the function of FOG-1 by sequestration such that it is unable to interact with GATA-1 and cooperate in the transcriptional regulation of GATA-1 target genes^56^. Bargo and colleagues illuminated that TACC3 binds to the intracellular domain of Notch receptor resulting in the inhibition of transcription of *Hey2*, a well-known target gene of Notch signaling^57^. On the other hand, TACC3 can also serve as a co-activator; for instance, Guo et al. and Partch et al. in tandem reported that TACC3 could bridge HIF and ARNT via its C-terminal coiled-coil domain, which is a necessary step for transcriptional responses to hypoxia^58,59^. Herein, we clarified that TACC3 promotes the mRNA expression of *E2F1*, which is supposed to be mediated by transcriptional activation or sequestration of the negative factors. Furthermore, we were able to reveal that TACC3 binds to the *E2F1* promoter (nucleotides −1 to −220) as well as the importance of this promoter region in the regulation of *E2F1* promoter activity by TACC3. We further used TRANSFAC^60^, a powerful prediction tool, to search the potential transcription factors and found that E2F family proteins, specificity protein 1 (Sp1), and nuclear factors (NF-Y) were associated with this *E2F1* promoter region (Supplementary Fig. 3). The motif analysis revealed two sets of overlapping E2F-binding sites located close to the transcription initiation site. It has been shown that the distal E2F-binding site on the *E2F1* promoter is required for transcriptional repression, whereas the proximal E2F-binding site contributes to transcriptional activation^61^. We speculated that TACC3 may enhance E2F transcription activators such as E2F1, E2F2, and E2F3a and/or attenuate the binding of E2F transcription repressors including E2F3b, E2F4-8 to the *E2F1* promoter. NF-Y has also been shown to directly bind to the *E2F1* promoter and facilitate *E2F1* expression raising the possibility that TACC3 may increase its binding to the *E2F1* promoter and thus induce *E2F1* mRNA transcription^62^. In addition, the region of the *E2F1* promoter bound by TACC3 also contains mutiple consensus Sp1-binding sites (CCGCCC), and a study has shown that Sp1 cooperates with NF-Y to induce *E2F1* expression in MCF-7 and ZR-75 cell lines when treated with estradiol^63^. Therefore, it is possible that TACC3 may alter the regulation of *E2F1* transcription via more than one transcription factor. In future studies, we will identify transcription factors that are involved in TACC3-mediated *E2F1* regulation and the exact mechanism.

Noticeably, it has been previously claimed that TACC3 proteins may form a complex with different histone acetyltransferases, including hGCN5L2 and pCAF, directly implicating TACC3 in transcriptional control^64^. Two years later, Angrisano et al. reported that TACC3 interacts with MBD2 on methylated promoters and then forms a complex with histone acetyltransferases switching the MBD2-mediated transcriptional repression to activation without involving promoter demethylation^65^. Surprisingly, our preliminary data showed that suppression of TACC3 dramatically decreases the acetylation level of histone H3 on *E2F1* promoter region (data not shown). In general, these raise the possibility that aberrant expression of TACC3 may affect gene regulation through inducing epigenetic changes mediated by interaction with components of chromatin remodeling complexes, thus contributing to tumorigenesis.

Perioperative cisplatin-based combination chemotherapy is the current standard treatment for high-risk MIBC after radical cystectomy. However, this therapy is only effective in 30–40% of cases. According to woefully inaccurate staging systems that are currently used, it is not yet possible to prospectively select the patients who are likely to experience a therapeutic benefit. Therefore, the development of a more precise, biology-based approach to the classification of bladder cancer is urgently needed in order to improve clinical management of this disease. Notably, our results revealed that elevated levels of TACC3 or E2F1 increase cellular sensitivity to cisplatin, which is possibly due to an E2F1-mediated functional perturbation of the intracellular balance between certain pro-apoptotic (i.e., *Bik* and *Bim*) and anti-apoptotic (i.e., *Bcl-2*) components.

In summary, since TACC3 overexpression seems to indicate an aggressive phenotype, it is tempting to speculate that TACC3 would be a potential therapeutic target or a prognostic factor that could be used to predict therapeutic modalities and outcomes in bladder cancer.

## Materials and methods

### Clinical specimens

Fresh-frozen tissues from patients with bladder cancer who were histopathologically and clinically diagnosed and who did not undergo neo-adjuvant treatment were obtained from the Cancer Center of Sun Yat-sen University in Guangzhou. Written informed consent was obtained from each patient, and the study was approved by the Institute Research Ethics Committee at the Cancer Center.

### Cell lines

The bladder cancer cell lines HT1197, TCCSUP, T24, 5637, J82, EJ, and SW780 and the immortalized normal bladder epithelial cell line SV-HUC-1 were obtained from the American Type Culture Collection (ATCC, Manassas, VA, USA). Cancer cell lines were cultured in Dulbecco’s modified Eagle’s medium (DMEM; Gibco, Carlsbad, CA, USA) supplemented with 10% fetal bovine serum (FBS; Gibco), while SV-HUC-1 cells were cultured in F-12 medium with 10% FBS (Gibco). All cell lines were cultured in a humidified 5% CO_2_ incubator at 37 °C.

### Plasmids

Luciferase reporter gene constructs containing the *E2F1* promoter of 1.2-kb genomic fragment (WT) or deletion fragment 6 were first amplified by PCR with specific primers and then the PCR products were cloned into pGL3-basic (Promega) digested by xhoI and Hind III using ClonExpress II One Step Cloning Kit (C112-02, Vazyme Biotech).

### *E2F1*-WT

Sense: 5′-gcgtgctagcccgggctcgagGCCAGACCAGTGTTGGGAGG-3′

Antisense: 5′-cagtaccggaatgccaagcttCAAAGTCCCGGCCACTTTTA-3′

*E2F1*-delta Fragment 6:

Sense: 5′-gcgtgctagcccgggctcgagGCCAGACCAGTGTTGGGAGG-3′

Antisense: 5′-cagtaccggaatgccaagcttGTGACTCCCCGCCCCCAG-3′

### Quantitative RT-PCR analysis

Total RNA was extracted from the cultured cells and fresh tissues using TRIzol reagent (Invitrogen, Grand Island, NY, USA) according to the manufacturer’s instructions. The first strand of DNA (cDNA) was synthesized using a reverse transcriptase kit (Invitrogen). The expression data were normalized to the housekeeping gene actin, and the relative expression levels were calculated as follows: 2 ^Ct value of gene−Ct value of actin^, in which Ct represents the threshold cycle for each transcript. Three independent experiments were performed to verify the quantitation of the data. The following primers were used in the qRT-PCR assays:

TACC3

Sense: 5′-CCTCTTCAAGCGTTTTGAGAAAC-3′

Antisense: 5′-GCCCTCCTGGGTGATCCTT-3′

*E2F1*

Sense: 5′-GACGGCTTGAGGGGTTGAC-3′

Antisense: 5′-ATGCTACGAAGGTCCTGACAC-3′

*actin*

Sense: 5′-CGCGAGAAGATGACCCAGAT-3′

Antisense: 5′-GGGCATACCCCTCGTAGATG-3′

*Bim*

Sense: 5′-TGACTCTCGGACTGAGAAACG

Antisense: 5′-CCTTCAGGATTACCTTGTGGCT

*Bik*

Sense: 5′-TGGAGGTTCTTGGCATGACTG-3′

Antisense: 5′-TGAGGCTCACGTCCATCTCG-3′

*BCL2*

Sense: 5′-GTGGACAACATCGCCCTGTG-3′

Antisense: 5′-GCCAAACTGAGCAGAGTCTTCA-3′

### Public expression profiles

Expression profiles of Kim et al. and Lee et al. (GSE13507), Dyrskjøt et al. (GSE3167), Riester et al. (GDS4456, GSE31684), and Sjödahl et al. (GSE32894) were downloaded from GEO (https://www.ncbi.nlm.nih.gov/geo/). E-MTAB-1803 was downloaded from EMBL-EBI (http://www.ebi.ac.uk/). Box-and-whisker plots depict the median, the first and third quartiles, and the minimum and maximum. Statistical significance was assessed by Student’s *t*-test.

### Western blot analysis

Western blotting was performed as previously described. The primary and conjugated secondary antibodies used are as follows: TACC3 (Proteintech), P-AKT (Thr473)(Santa Cruz), AKT (CST), cyclin D1 (CST), E2F1 (Proteintech), actin (Abcam), tubulin (Sigma), Flag (M2, Sigma), and Myc (9E10, Santa Cruz).

### siRNA transfection

siRNA oligo-ribonucleotides of *TACC3* were purchased from RiboBio (RiboBio, Guangzhou, China) and had the following target sequence:

a. ACTAGATTGCTTTGAAAAC

b. CCACAGATCTGAACTCCAT

EJ and 5637 cells (2 × 10^5^) were seeded into 6-well plates, incubated for 24 h, and then transfected with 40 nM of the RNA duplex and 5 μl of Lipofectamine RNAiMAX (Invitrogen, Grand Island, NY, USA) according to the manufacturer’s instructions. The cells were harvested for further experiments after 48 or 72 h.

### MTT assay

An MTT assay (Sigma, St Louis, MO, USA) was used to measure cell growth according to the manufacturer’s instructions. Briefly, approximately 2000 cells were seeded in 96-well plates and cultured in DMEM supplemented with 10% FBS. Cell proliferation was examined on 1, 2, 3, 4, 5, and 6 days according to standard procedures. All experiments were repeated three times.

### Colony formation assay

After knockdown or overexpression of TACC3, bladder cancer cells were plated in triplicate in 6-well plates at a density of 1000 cells per well and cultured for 15 days. After fixation in methanol for 15 min, the colonies were stained with 0.5% crystal violet in 20% methanol for 15 min and counted. All experiments were repeated three times.

### Cell migration assay

The cell migration assay was performed using Transwell chambers (Costar, Corning Inc.). The bottom chamber of the Transwell device was filled with 700 ml of DMEM supplemented with 10% FBS, while the cells (5 × 10^4^) were seeded in the top chamber. After 24 h of incubation, the cells that had invaded the bottom of the inserts were fixed, stained, photographed, and quantified by counts in five random high-magnification fields.

### Cell-cycle analysis

Both EJ and 5637 cell lines were transfected with control siRNAs and TACC3 siRNAs. After 48 h, the cells were trypsinized and fixed in ice-cold 70% ethanol. The cells were subsequently stained with Propidium Iodide (PI) for cell-cycle analysis (Sigma, USA). Cells were analyzed for DNA content by flow cytometry using a Cytomics FC 500 (Beckman Coulter, Fullerton, CA, USA). The data were analyzed using FlowJo 7.6 software (Beckman).

### G1/S transition assay

Bladder cancer cell lines that stably express TACC3 were treated with nocodazole (100 ng/ml, Sigma-Aldrich, St Louis, MO, USA) for 16 h. Cells were harvested by trypsinization and fixed in ice-cold 70% ethanol overnight. Cell-cycle distributions were measured by staining with propidium iodide, which was followed by flow cytometric analysis (Beckman).

### Dual luciferase reporter assay

A dual luciferase reporter assay was performed as previously described with slight modifications. Briefly, the treated bladder cancer cells were lysed, and the activities of the firefly and Renilla luciferases were analyzed using a dual luciferase assay kit (Promega, Madison, WI, USA) according to the manufacturer’s instructions. All reporter gene assays were performed in triplicate and were repeated twice. The results are expressed as mean ± SEM.

### Chromatin immunoprecipitation (ChIP) assays

ChIP assays were performed using a ChIP assay kit (Upstate Biotechnology, Lake Placid, NY, USA) according to the manufacturer’s instructions. Immunoprecipitation was performed using antibodies specific for TACC3 or with rabbit IgG. Purified ChIP DNA was subjected to qRT-PCR with the following promoter-specific primer sets:

Fragment 1(-1258--1077)

Sense: 5′-CAGACCAGTGTTGGGAGGAG-3′

Antisense: 5′-AACACATCACTTCTAGGGAGCC-3′

Fragment 2(-1076--809)

Sense: 5′-AGGATCTCGCTCTGTCGCC-3′

Antisense: 5′-GCCTGATCAACATGGTGAAGAC-3′

Fragment 3(-808--551)

Sense: 5′-AGACGCCACTTCATCGTATTG-3′

Antisense: 5′-CAAGTCCCATTTTAGCCAAAAT-3′

Fragment 4(-550--404)

Sense: 5′-GGGTGAGCGGGATAGGG-3′

Antisense: 5′-CGTGACCCTCAACCTGTAGC-3′

Fragment 5(-403--221)

Sense: 5′-TGTTGGGGGCTACAGGTTG-3′

Antisense: 5′-CAGGCGCGGTGTGACTC-3′

Fragment 6(-220--1)

Sense: 5′-TCCGGACAAAGCCTGCG-3′

Antisense: 5′-TCCCGGCCACTTTTACGC-3′

Each ChIP assay was repeated three times, and a representative result is presented.

### BrdU incorporation and immunofluorescence

BrdU was prepared according to standard methods. Briefly, cells were grown on glass coverslips and were incubated for 1.5 h with culture media containing BrdU (Amersham Biosciences, Bucks, UK) at a dilution of 1:10,000 (10 mM). All cells were fixed in 70% ethanol and washed, and the BrdU-treated cells were incubated for 10 min with 2 M HCl. An anti-BrdU (Abcam Ltd, Cam, UK) primary antibody and an anti-mouse fluorescein-5-isothiocyanate (FITC)-conjugated secondary antibody (Molecular Probes, Eugene, OR, USA) were used.

### Statistical analysis

All statistical analyses were performed using the SPSS 16.0 statistical software package (SPSS Inc, Chicago, IL, USA). Data are shown as mean ± SEM. Categorical data were tested by the *χ*^2^ test. Survival curves were plotted using the Kaplan–Meier method and were compared by the log-rank test. Other comparisons were performed using an unpaired two-sided Student’s* t*-test or one-way ANOVA; *p* < 0.05 was considered statistically significant; *p < 0.05, **p < 0.01, ***p < 0.001.

## Electronic supplementary material

Supplemental information
