# Supplementary material for: TACC3 transcriptionally upregulates E2F1 to promote cell growth and confer sensitivity to cisplatin in bladder cancer
Source: Cell Death Dis. 2018 Jan 22;9(2):72. doi: 10.1038/s41419-017-0112-6 (PMC5833822; doi:10.1038/s41419-017-0112-6)
Supplement: Supplementary file 1 — Supplemental information [file 41419_2017_112_MOESM1_ESM.docx]

**Supplemental information**


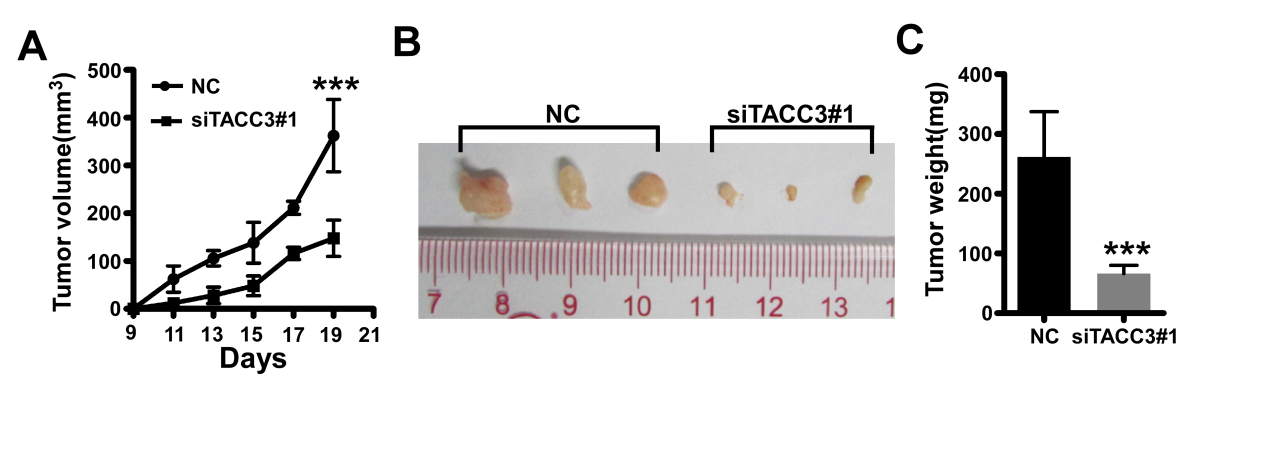


**Supplementary Figure 1 Suppression of TACC3 dramatically inhibited tumor growth and proliferation in vivo as determined by a subcutaneous xenograft mice model**

(A) The mean volume of he tumours.

(B) Actual sizes of representative tumours.

(C) The weight of the tumours.

Results were presented as mean s.e. (n =3 tumours). The significance were calculated by the Student’s t-test. *** means p<0.001.





**Supplementary Figure 2 TACC3 affects E2F1's direct target genes such as *MCM4*, *CDC6*, and *CDC25A***

(A) Inhibition of the TACC3 expression by siRNAs could repress the expression of three E2F1-targeted genes, including *MCM4*, *CDC6* and *CDC25A*. Error bars present mean ± SEM (n=3). The p values were calculated by one-way ANONA. *** means p<0.001.

(B) TACC3 overexpression enhanced the expression of *MCM4*, *CDC6* and *CDC25A*. Error bars present mean ± SEM (n=3). The p vlaues were calculated by the Student’s t test. *** means p<0.001.

(C) Scatter plots shown *TACC3* mRNA level relates to the mRNA levels of *MCM4*, *CDC6* and *CDC25A*. X axis indicates *TACC3* RPKM value, Y axis indicates the RPKM value of *MCM4*, *CDC6* and *CDC25A*, respectively. The p value and R value were calculated by Pearson correlation coefficient.

**
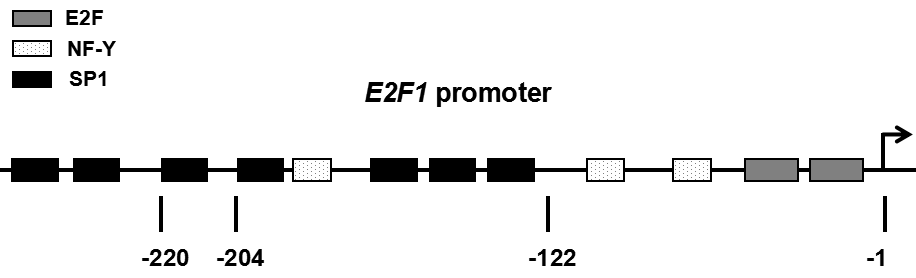
**

**Supplementary Figure 3 Schematic depicting regulatory elements within the *E2F1* promoter**

Consensus Spl-binding sites (CCGCCC) and NF-Y response element (CCAAT boxes) are indicated as well as E2F-binding sites.
